# A single institution’s experience with minimally invasive surgery for ovarian cancer, and a systematic meta-analysis of the literature

**DOI:** 10.1007/s10147-023-02320-2

**Published:** 2023-04-28

**Authors:** Yuji Kamei, Eiji Kobayashi, Eiji Nakatani, Mayu Shiomi, Masaaki Sawada, Mamoru Kakuda, Aska Toda, Satoshi Nakagawa, Kosuke Hiramatsu, Yasuto Kinose, Tsuyoshi Takiuchi, Ai Miyoshi, Michiko Kodama, Kae Hashimoto, Toshihiro Kimura, Yutaka Ueda, Kenjiro Sawada, Tadashi Kimura

**Affiliations:** 1 Department of Obstetrics and Gynecology, Osaka University Graduate School of Medicine, 2-2, Yamadaoka Suita, Osaka, 567-0871 Japan; 2 Division of Statistical Analysis, Research Support Center, Shizuoka General Hospital, Shizuoka, Japan; 3 Department of Obstetrics and Gynecology, Oita University Graduate School of Medicine, 1-1, Hasamamachiidaigaoka Yufu, Oita, 879-5503 Japan

**Keywords:** Laparoscopy, Laparotomy, Minimally invasive surgery, Ovarian cancer

## Abstract

**Background:**

This study assesses the feasibility of minimally invasive surgery (MIS) for well-selected epithelial ovarian cancer (EOC) patients.

**Methods:**

We performed a review of data prospectively collected from a single center from 2017 to 2022. Only patients with histologically confirmed EOC, with a tumor diameter of less than 10 cm, were eligible. We also performed a meta-analysis of similar studies comparing the outcomes of laparoscopy and laparotomy. We used MINORS (Methodological Index for Non-Randomized Studies) to assess the risk of bias and calculated the odds ratio or mean difference.

**Results:**

Eighteen patients were included; 13 in re-staging group, four in PDS group, and one in IDS group. All achieved complete cytoreduction. One case was converted to laparotomy. The median number of removed pelvic lymph nodes was 25 (range 16–34), and 32 (range 19–44) for para-aortic nodes. There were two (15.4%) intraoperative urinary tract injuries. The median follow-up was 35 months (range 1–53). Recurrence was observed in one case (7.7%). Thirteen articles for early-stage ovarian cancer were included in our meta-analysis. Analysis of the pooled results found that MIS had a higher frequency of spillage (OR, 2.15; 95% CI 1.27–3.64). No differences were observed in recurrence, complications, or up-staging.

**Conclusions:**

Our experience supports the possibility of conducting MIS for EOC in well-selected patients. Except for spillage, our meta-analysis findings are consistent with previous reports, the majority of which were also retrospective. Ultimately, randomized clinical trials will be needed to authenticate the safety.

**Supplementary Information:**

The online version contains supplementary material available at 10.1007/s10147-023-02320-2.

## Introduction

Surgery for ovarian cancer, which can include fallopian tube, peritoneal, and borderline malignant ovarian tumors, is mainly performed by laparotomy. Laparotomy is one of the most invasive treatments in gynecology, frequently resulting in complications such as ileus and infection [1], and in some cases, postoperative treatment may be delayed [2]. There are only limited reports on the use of laparoscopic minimally invasive surgery (MIS) for ovarian cancer in Japan [3, 4]. Since the first reports of MIS experiences in 1994 [5], there have been many such reports published in other countries [6–8]. Compared with laparotomy, laparoscopy is less invasive and is associated with less postoperative pain, intraoperative blood loss, and a shorter length of hospitalization [6], and it is believed to have a significant positive effect on the patient's quality of life (QOL) [9].

The required staging procedure for laparoscopy is the same as that used for uterine cancer, so MIS can be performed in the same manner for ovarian cancer in well-selected patients. Analysis of data from the National Cancer Data Base has also suggested that laparoscopic staging of apparent early-stage (EOC) epithelial ovarian cancer is safe—when performed by a surgeon with appropriate training [10, 11].

A recent meta-analysis found that patients diagnosed with early-stage or advanced-stage ovarian cancer undergoing MIS presented similar three-year and five-year recurrences and mortalities [11]. However, there are some cases, such as those with massive ovarian cancer or with multiple peritoneal dissemination that can only be performed by laparotomy. Still, we believe that laparoscopic surgery can be safely performed in carefully selected patients by a surgeon with appropriate training.

Since there have been only limited reports of this nature in Japan, the purpose of our study was to evaluate the feasibility of laparoscopic surgery, in terms of safety and efficacy, in patients with multiple forms of ovarian cancer, as a single institution experience. Furthermore, we have put our institution’s experiences concerning MIS for ovarian cancer into perspective by reviewing the relevant recent literature from other countries.

## Materials and methods

### Study design and population of our original retrospective study

We reviewed prospectively collected data from surgeries conducted from September 2017 to April 2022 at our hospital, Osaka University Hospital. Only patients with histologically confirmed EOC were eligible for this study. We divided the eighteen eligible EOC patients into three groups: (1) the primary debulking surgery (PDS) group, patients with preoperatively stage I ovarian cancer, with a tumor diameter of less than 10 cm (including fallopian tube, peritoneal, or borderline malignant ovarian tumors); (2) a re-staging (RS) group, in which the patient was incidentally diagnosed with a borderline malignant or malignant ovarian tumor, which had been initially suspected of being benign before surgery, and additional staging surgery was needed; (3) and an interval debulking surgery (IDS) group, in which cases with advanced-stage ovarian cancer showed a response to neoadjuvant chemotherapy.

Further study eligibility criteria included the following: the patient need to have an Eastern Cooperative Oncology Group (ECOG) performance status of 0–2; adequate major organ function, with an absolute neutrophil count of > 1500/mm^3^, platelet count of 100,000/mm^3^ or higher, AST/ALT less than 100 IU/L, total serum bilirubin less than 1.5 mg/dl, serum creatinine less than 1.5 mg/dl, and electrocardiography within normal limits—or asymptomatic and not requiring treatment) (all laboratory tests must have been performed within 28 days before the scheduled surgery), and at least 20 years of age or older at the time of enrollment. Some patients had undergone an initial surgery at a different hospital, then were subsequently admitted and underwent chemotherapy and/or surgery at our institution.

Patients were not considered eligible when any of the following criteria were present: the patient had serious morbidities or complications (i.e., serious heart or cerebrovascular disease, diabetes with HbA1c > 8.5%, difficult to control hypertension, pulmonary fibrosis, interstitial pneumonia, hemorrhage, active peptic ulcer, or serious neurological disease. Patients were also excluded who were expected to have difficulty in completing this study or subsequent follow-up, or who were judged by their physician to be inappropriate for participation.

### Collected data of the original study

We recorded the following patient characteristics: age, body mass index (BMI), ECOG performance status, tumor size, FIGO clinical stage, and tumor histology. The clinical response to neoadjuvant chemotherapy was evaluated by assessing CA-125 serum levels and CT scans according to the Response Evaluation Criteria in Solid Tumors (RECIST) criteria.

We recorded the following intraoperative parameters: the degree of residual disease, operative time, blood loss, need for transfusion, and any surgical complications. Postoperative parameters included any unplanned readmission within 30 days of surgical discharge. Moreover, the number of removed lymph nodes, pathological response, length of stay, median follow-up duration, recurrence, and disease-free survival were obtained.

Post-surgery gynecological examinations, pelvic ultrasonography, and CA-125 serum levels were performed every three months, and CT scans every 6 months.

### Surgical procedure of the original study

All surgical teams included at least one qualified gynecologist certified by the Endoscopic Surgical Skill Qualification System in Japan. A careful exploration of the peritoneal cavity was first conducted, using a 10 mm 0° laparoscope at the umbilical site. ‘Standard cytoreduction’ consisted of a hysterectomy, bilateral salpingo-oophorectomy, and omentectomy. Peritonectomy, pelvic and aortic lymphadenectomy, or other abdominal procedures (i.e., anterior rectal resection) were performed, depending on the patient. A specimen bag was used to retrieve the lymph nodes, peritoneum, and omentum. Complete cytoreductive surgery was defined as when all visible tumors were removed by the end of the surgery.

### Meta-analysis

**Fig. 1:**
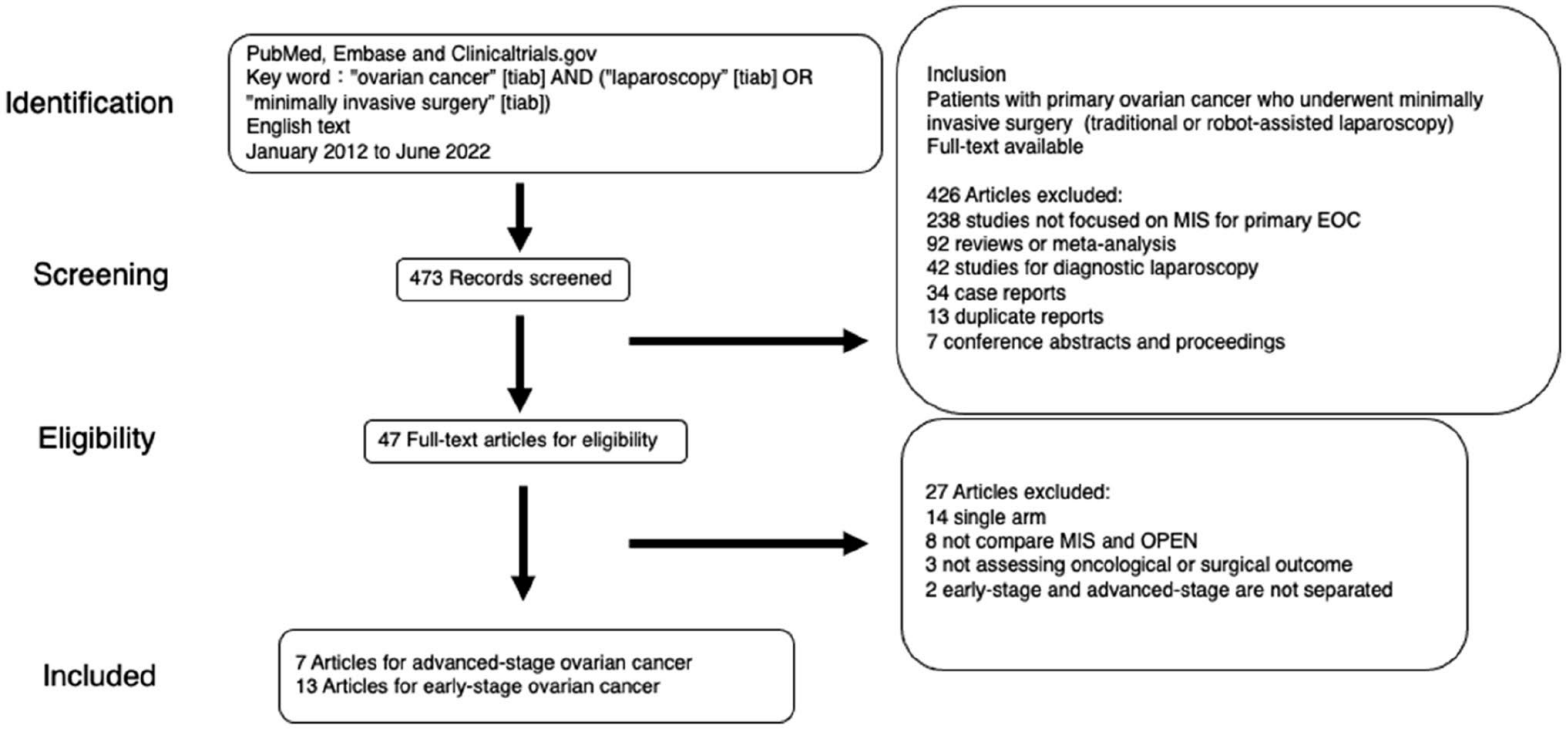
Flow diagram of the research selection progress

We planned and conducted a meta-analysis of the literature according to Preferred Reporting Items for Systematic Review and Meta-Analysis (PRISMA) guidelines. Eiji Nakatani, who is our co-author and a biostatistician, was involved in the design and conduction of our meta-analysis. The search process is shown in Fig. 1. We performed an English-language literature search of papers in PubMed (https://pubmed.ncbi.nlm.nih.gov), Embase (https://www.embase.com), and Clinicaltrials.gov, published from January 2012 to June 2022, using the required keywords “ovarian cancer” [tiab] and (“laparoscopy” [tiab] or “minimally invasive surgery” [tiab]). We screened articles on each database without using any online tools. Articles comparing the ovarian cancer outcomes of laparoscopy (including robot-assisted laparoscopy) and laparotomy were considered appropriate for analysis. We initially identified 473 potential records. Available full-text studies of patients with primary ovarian cancer who underwent MIS were included. For eligibility, studies that were not focused on MIS, reviews or meta-analysis, or studies for diagnostic laparoscopy, and case reports, duplicate reports, conference abstracts and proceedings were excluded—after reading the titles and abstracts. After applying all inclusion and exclusion criteria, 47 full-text articles remained. Finally, single-arm studies, studies not assessing oncological or surgical outcome, studies including both early and advanced-stage cancers, and studies not comparing laparoscopy and laparotomy were excluded from reading the full text. Thirteen articles for early-stage and seven articles for advanced-stage ovarian cancer were included in our meta-analysis. All search processes were reviewed by two independent reviewers (YK and EK), who discussed and settled any differences in their interpretations. In each report, the following data were extracted: age, BMI, stage, histology, adjuvant chemotherapy, operative time, blood loss, transfusion, spillage, up-staging, conversion to laparotomy, intra- and postoperative complications, number of removed lymph nodes, length of stay, residual tumor, follow-up, recurrence, port-site metastasis, and OS and PFS. We synthesized surgical-related outcomes (operative time, blood loss, transfusion, spillage, up-staging, operative complications, length of stay) and oncologic outcomes (recurrence) if there was no heterogeneity between studies.

### Statistical analysis

All statistical analyses were performed with EZR, in the free software environment R for biostatistics computing and graphics (version 1.55; available on CRAN as the “RcmdrPlugin.EZR” package). We assessed heterogeneity using the I^2^ statistic. If the I^2^ was > 50%, we assumed that heterogeneity was present. The random-effect model was adopted to allow for variability between studies. Studies with missing values for each parameter were excluded. In cases of heterogeneity or small patient numbers, results were not synthesized. We calculated the odds ratio (OR) or mean difference according to the specified parameters. The risk of bias was evaluated using the Methodological Index for Non-Randomized Studies (MINORS) [12]. Publication bias was assessed by creating a funnel plot.

### Ethics statement

This study was approved by the Institutional Review Board and the Ethics Committee of the Osaka University Hospital (#15568–9) and was conducted following all relevant guidelines and regulations.

## Results

### Our single institutional experience

Eighteen of the ovarian cancer patients met the criteria to be included in our study. Supplementary Fig. 1 shows a flowchart of the patient selection protocol. Table 1 shows the characteristics of each assigned patient treatment group. Thirteen cases were in the RS group, four were in the PDS group, and one was in the IDS group. The median age of the three groups was 49, 53, and 71 years, respectively; the median BMI was 22.1, 23.3, and 19.2 kg/m^2^, respectively.

**Table 1:** Patients characteristics

	Re-staging	PDS	IDS
	*N* = 13	*N* = 4	*N* = 1
Age (years)	49 (31–73)	53 (38–68)	71
BMI (kg/m^2^)	22.1 (16.3–26.3)	23.3 (20.5–27.5)	19.2
ECOG performance status			
0	13 (100)	4 (100)	1
1 or more	0	0	0
Diagnosis			
High grade serous	3 (23.1)	0	1
Mucinous	3 (23.1)	0	0
Clear cell	2 (15.4)	1 (25.0)	0
Endometrioid	2 (15.4)	1 (25.0)	0
Seromucinous borderline	1 (7.7)	2 (50.0)	0
Anaplastic	1 (7.7)	0	0
Malignant transformation of mature cystic teratoma	1 (7.7)	0	0
FIGO2014			
IA	4 (30.8)	3 (75.0)	0
IB	1 (7.7)	0	0
IC1	4 (30.8)	0	0
IC2	2 (15.4)	0	0
IC3	1 (7.7)	1 (25.0)	0
IIIA1	1 (7.7)	0	1
Tumor size			
No	13 (100)	0	0
0–4.9 cm	0	1 (25.0)	1
5–9.9 cm	0	3 (75.0)	0
Neoadjuvant chemotherapy cycles	NA	NA	4
RECIST response			
CR	NA	NA	0
PR			1
SD, PD			0
Adjuvant chemotherapy			
No	4 (30.8)	2 (50.0)	0
Carboplatin-paclitaxel	8 (61.5)	2 (50.0)	0
Carboplatin-paclitaxel-bevacizumab	1 (7.7)	0	1

Data are median (range) or *n* (%) unless otherwise specified

One of the 13 women in the RS group was found to have pelvic lymph node metastasis; her FIGO stage was upgraded accordingly. After four cycles of neoadjuvant chemotherapy, one patient in the IDS group had a partial response, although there was no peritoneal dissemination and only a small tumor in the ovary on imaging.

The operative data are shown in Table 2. The median operative time for the three groups was 421 min (range 215–580), 355 min (range 98–504), and 461 min, respectively. The estimated blood loss was 40 (range 20–1110), 115 (range 20–180), and 300 mL, respectively. In the RS group, all cases were postoperatively diagnosed as malignant. After the re-staging surgery, standard cytoreduction was achieved in all patients.

**Table 2:** Surgical data

	Re-staging	PDS	IDS
	*N* = 13	*N* = 4	*N* = 1
Method			
Hysterectomy	8 (61.5)	2 (50.0)	1
Salpingo-oophorectomy	5 (38.5)	4 (100)	1
Pelvic lymphadenectomy	12 (92.3)	2 (50.0)	1
Aortic lymphadenectomy	12 (92.3)	2 (50.0)	1
Omentectomy	11 (84.6)	3 (75.0)	1
Appendectomy	0	1 (25.0)	0
Peritonectomy	0	0	1
Intraoperative ovarian rupture	0	1 (25.0)	0
Residual disease			
R0	13 (100)	4 (100)	1
R1 or R2	0	0	0
Lymph nodes			
Pelvic	25 (16–34)	29 (24–34)	27
Aortic	29 (19–48)	31 (26–35)	30
Lymph node metastasis			
Pelvic	0 (0–5)	0	0
Aortic	0	0	3
Operative time (minute)	421 (215–580)	355 (98–504)	461
Estimated blood loss (mL)	40 (20–1110)	115 (20–180)	300
Blood transfusion			
No	12 (92.3)	4 (100)	0
Autologous	1 (7.7)	0	0
Allogeneic	0	0	1
Complication			
Intraoperative			
Ureter injury	1 (7.7)	0	0
Bladder injury	0	0	1
Postoperative			
Chylous ascites	1 (7.7)	0	0
Pelvic infection	1 (7.7)	0	0
Clavien–Dindo classification			
I or II	2 (15.4)		
III or IV	0		
Length of admission (day)	12 (7–14)	12 (7–15)	12
Unplanned readmission within 30 days of discharge	1 (7.7)	0	0

Data are median (range) or *n* (%) unless otherwise specified

In the PDS group, we performed an appendectomy on one of the four patients diagnosed with a mucinous borderline tumor via an intraoperative frozen section. One patient had previously undergone a hysterectomy due to a leiomyoma. One patient’s choice was to not have a hysterectomy and omentectomy performed, so as to preserve her fertility. In our study, all 18 patients reached complete cytoreduction. One case was converted to laparotomy due to suspected diffuse residual disease after the primary surgery. One cystic rupture was observed, but the case was not upstaged due to positive ascites cytology originally.

Histological findings in the IDS patient showed that some microscopic residual disease was found in the adnexa. The median number of removed pelvic and para-aortic lymph nodes was 25 (range 16–34) and 32 (range 19–48), respectively. One RS patient and one IDS patient had metastasis found during the pathological exam of the nodes.

There were two intraoperative complications. One patient in the RS group had an injury to the left ureter, so a catheter was inserted. This was a case of a second surgery, and because of endometriosis and strong fibrosis of the retroperitoneum, the ureter could not be adequately identified, resulting in a thermal injury to the ureter due to a misidentification. A different patient, in the IDS group, with multiple tiny tumors on the Douglas pouch and vesicouterine peritoneum, had a bladder injury in the process of stripping the peritoneum of the vesicouterine fossa; the injury was repaired with sutures. Two postoperative complications were registered in the RS group. In the first, chylous ascites was treated with a low-fat diet. The other case had a pelvic infection that was treated with antibiotics.

Nine patients in the RS group, two in the PDS group, and the one IDS patient underwent adjuvant chemotherapy. Eleven of these 12 patients completed their adjuvant chemotherapy. One patient discontinued chemotherapy after developing an intraperitoneal abscess in the 4th cycle. Of the six patients who omitted adjuvant chemotherapy, three had a borderline tumor, the other three were stage IA cases.

Table 3 shows the follow-up data from the 18 patients treated at our institution. The median follow-up in this study to date was 35 months (range 1–53). One peritoneal recurrence was observed in the RS group, with a DFS of 8 months; after her third-line chemotherapy, she died of the disease, with an OS of 18 months. The other 17 patients are all alive and free from recurrence.

**Table 3:** Follow-up

	Re-staging	PDS	IDS
	*N* = 13	*N* = 4	*N* = 1
Follow up period (month)	20 (5–53)	43 (1–48)	53
Disease free survival (month)	20 (5–53)	43 (1–48)	53
Recurrence			
No	12 (92.3)	4 (100)	1
Yes	1 (7.7)	0	0
Status			
NED	12 (92.3)	4 (100)	1
DOD	1 (7.7)	0	0

Data are median (range) or *n* (%) unless otherwise specified

### Meta-analysis

#### Screening of studies and selection

We show the data collected from the relevant studies in Supplementary Table 1 and 2. The number of studies where laparoscopy was used for advanced-stage disease was understandably small, so no meta-analysis was performed on them.

#### Summary of the selected studies

**Fig. 2:**
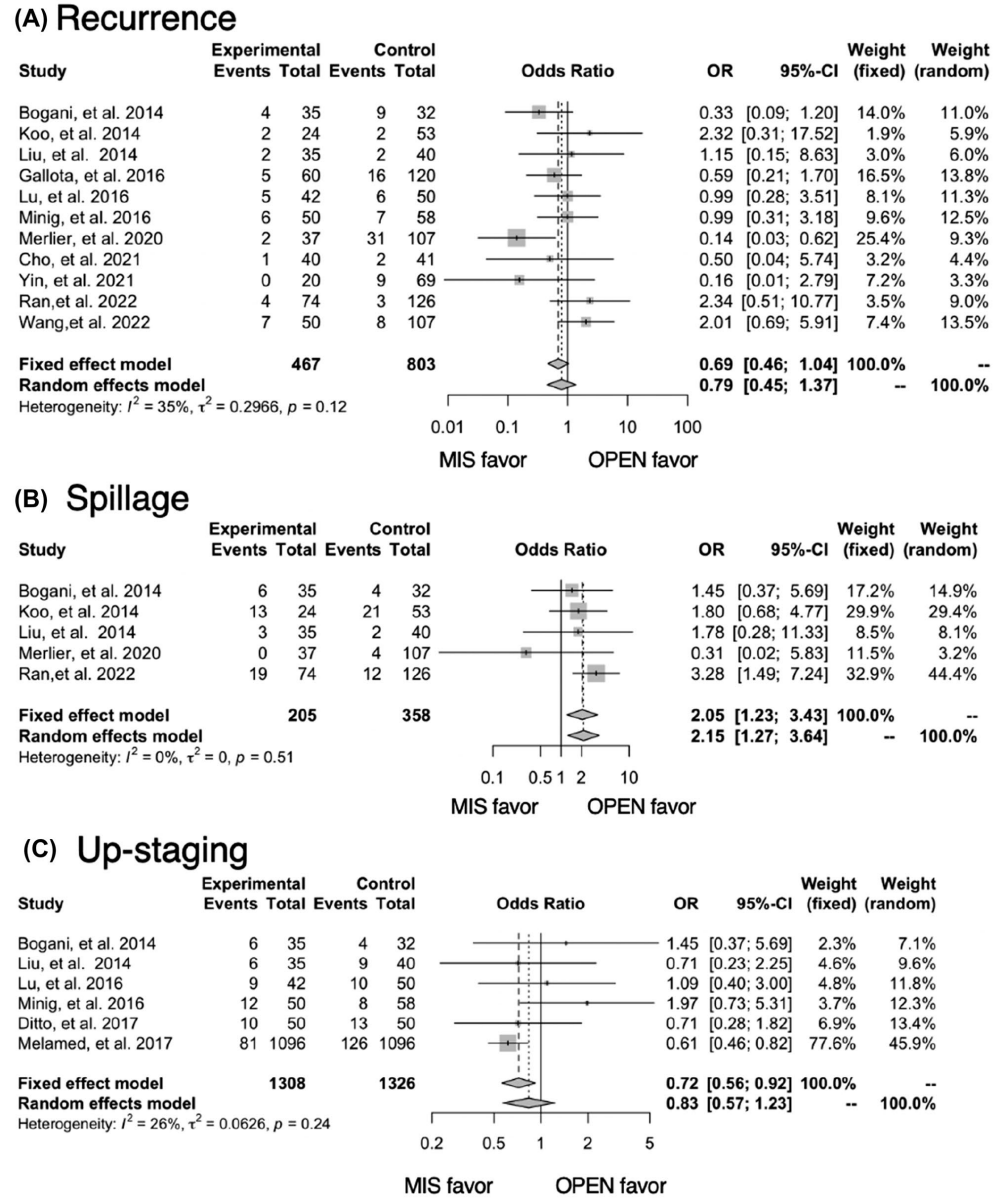

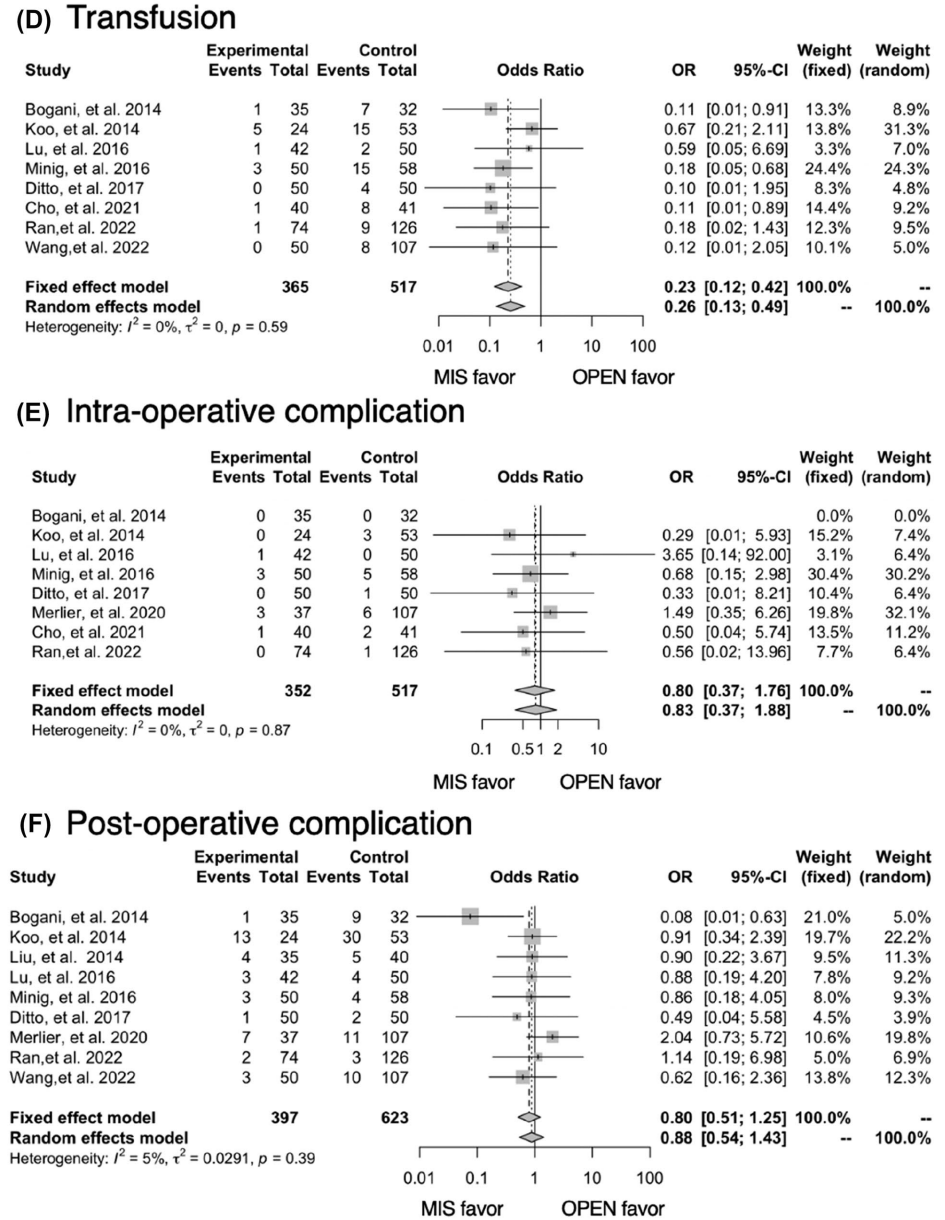
Meta-analysis of outcomes for early-stage ovarian cancer: MIS vs OPEN. Pooled analysis: **A** recurrence, **B** spillage, **C** up-staging, **D** transfusion, **E** intra-operative complication, **F** Post-operative complication

The forest plots comparing the outcomes between laparoscopic MIS and traditional laparotomy (OPEN) surgery are displayed in Fig. 2.A pooled analysis of the included studies found that MIS had less need for a transfusion (OR 0.26; 95% CI 0.13–0.49), but a higher frequency of spillage (OR 2.15; 95% CI 1.27–3.64). No differences were observed in recurrence rates (OR 0.79; 95% CI 0.45–1.37), intra-operative (OR 0.83; 95% CI 0.37–1.88) or postoperative complications (OR 0.88; 95% CI 0.54–1.43), and up-staging (OR 0.83; 95% CI 0.57–1.23). Operative times were not integrated due to the heterogeneity in reporting (*I*^2^ = 93%). Blood loss (*N* = 4) and length of hospital stay (*N* = 3) were not integrated due to the small number of papers where this was reported.

#### Subgroup analysis

Among the pooled analysis of recurrence rates, three of the studies showed significantly shorter follow-up period in MIS (Supplemental Table 1). We performed a subgroup analysis of recurrence rates, excluding three studies. No differences were observed in recurrence rates (OR 1.12; 95% CI 0.66–1.89) (Supplementary Fig. 2).

#### Quality assessment and heterogeneity among the studies

**Fig. 3:**
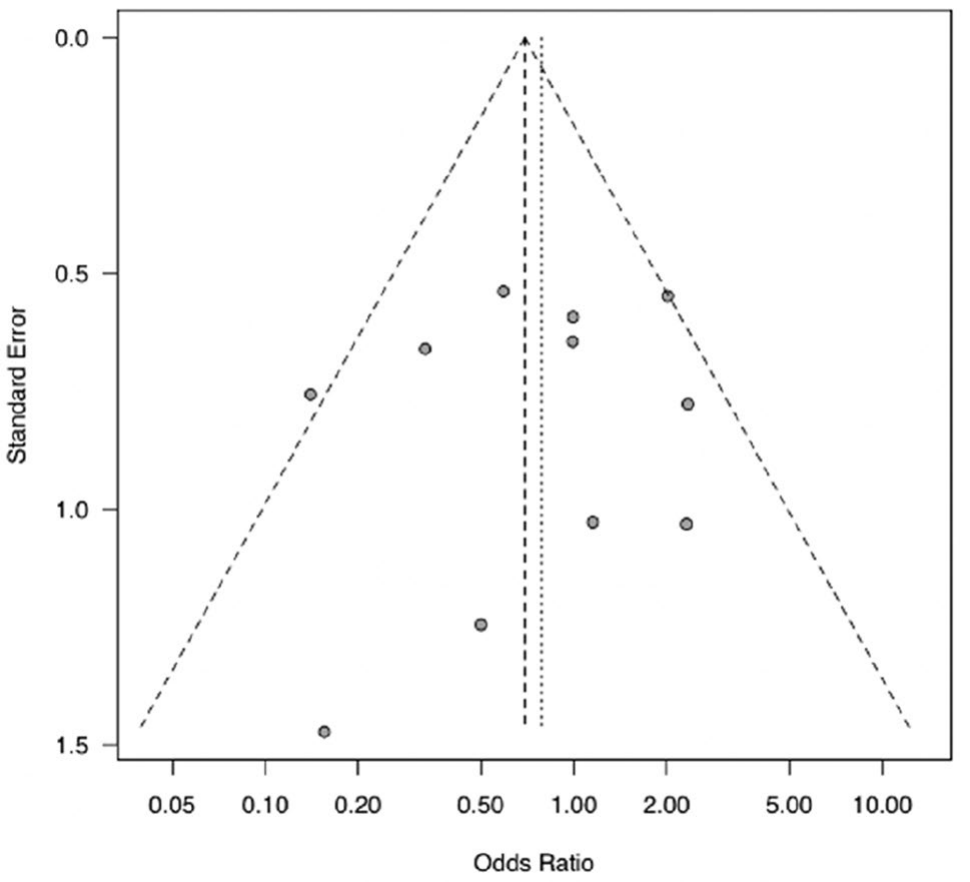
A funnel plot showing tumor recurrences in the eleven studies used for meta-analysis

The median MINORS score was 17 out of 24 (range 14–19). The funnel plot of the studies included in this meta-analysis indicated there was no obvious bias in the publications (Fig. 3). Heterogeneity among the studies was estimated to be low (*I*^2^ < 50%) (Fig. 2).

## Discussion

Our single-center experience suggests that the use of MIS in well-selected EOC cases is possible, although urinary tract injury should be noted in re-operative and post-NAC cases. Seventeen of 18 surgeries reached complete cytoreduction without conversion to a laparotomy. In terms of patient safety, two lower urinary tract injuries occurred during MIS for EOC (15.4%), but they had no apparent sequelae. Lower urinary tract injuries are a common complication of gynecological surgery and sometimes worsen the patient’s QOL. For benign conditions, the rate of urinary injury is quite low (< 1%), but in ovarian cancer, the rate is up to 4% [13]. Laparoscopic urinary tract injuries were relatively more common in our MIS study, 15.4%, suggesting that there may be room for improvement.

Our single-institutional experience is admittedly limited; still, we need to carefully note intraoperative complications because many ovarian cancer surgeries are remedial, i.e., performed either for the second time or after chemotherapy.

Recently, in a meta-analysis comparison of MIS invasive versus open surgery, Jochum [11] reported that the three and five-year mortality and recurrence rates and surgical outcomes in early and advanced-stage ovarian cancer were similar for laparoscopy and laparotomy. Most of our results were consistent with theirs. However, the majority of meta-analyses studies, and ours, were retrospective. Therefore, we must keep in mind the possibility of publication bias, which can be reflected in reports of fewer complications and better prognosis compared to actual clinical practice. To reach a definitive conclusion on the safety of laparotomy for ovarian cancer, future randomized clinical trials should be given a high priority. We are now awaiting the results of the LANCE study [14], a phase III trial comparing MIS with laparotomy in women with advanced ovarian cancer who had a complete or partial response to their neoadjuvant chemotherapy.

Currently, there are two major concerns with MIS compared to traditional open laparotomy. First, there is a concern that spillage of tumor cells during laparoscopic surgery is more likely to occur. Romagnolo [15] reported that the incidence of spillage was greater in laparoscopy than in laparotomy. In a large observational study conducted in the United States, MIS was associated with an increased risk of rupture [16]. Our meta-analysis showed similar results. However, meta-analyses conducted by others disagree, finding that there was no significant difference between laparoscopy and laparotomy [17, 18]. A review written for the Cochrane Library concluded that staging of early ovarian cancer by laparoscopy was technically feasible—when it was performed by experienced oncology surgeons [18].

There have been several reports on the impact of ovarian tumor rupture on disease-free survival. In a recent study, tumor rupture was associated with increased mortality [16]. Furthermore, a systematic review and meta-analysis of high-quality observational studies found that rupture resulted in decreased progression-free survival and overall survival [19].

Because a larger tumor size is associated with a higher risk of rupture [16], MIS should be limited to selected patients with small early-stage ovarian tumors. Because the isolation bag we used in this study was limited in use to tumors of 10 cm, we generally limit the surgery to cases with tumors of less than 10 cm by preoperative examination. Although further studies are needed, we believe the criterion for selecting a tumor diameter of less than 10 cm is technically reasonable.

A second concern with laparoscopy for EOC is the possibility of port-site metastases (PSM). In large studies, the frequency of PSM has ranged 2.3–46.7% [20]. PSM is thus a rare event in laparoscopy used for early-stage disease and does not seem to have a significant impact on survival [21]. PSM may be a surgical-technique-related issue that is limited mostly to patients with advanced disease [22], as no PSM has been reported in stage I ovarian cancers. In 2003, Ramirez described in great detail their highly successful methods for PSM prevention [23].

Our single-institutional study have its limitations, including the low number of cases (18) reported. However, our study supports the possibility of using MIS for early-stage ovarian cancer. We believe that carefully selecting the right patients and using only properly skilled surgeons is essential for conducting reduced-risk MIS for EOC. To definitively prove this belief, we will need the results of large-scale randomized clinical trials.

### Other information

The meta-analysis in this study was not registered. The templates for the data collection forms and the data used for all analyses are available from the corresponding author upon reasonable request.

## Supplementary Information

Below is the link to the electronic supplementary material.

Supplementary file1 (DOCX 319 KB)

Supplementary file2 (DOCX 62 KB)

## Data Availability

The data that support the findings of this study are available on request from the corresponding author.
