# Supplementary material for: A single institution’s experience with minimally invasive surgery for ovarian cancer, and a systematic meta-analysis of the literature
Source: Int J Clin Oncol. 2023 Apr 28;28(6):794–803. doi: 10.1007/s10147-023-02320-2 (PMC10232596; doi:10.1007/s10147-023-02320-2)

**Supplementary Fig. 1** A Flow chart of the patient selection protocol


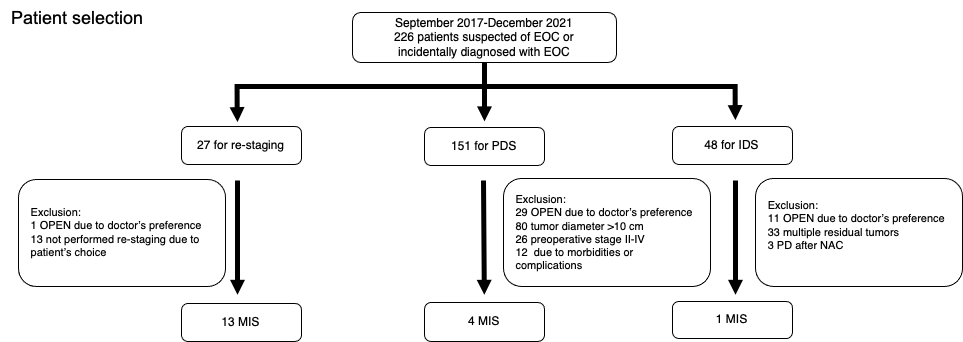


**Supplementary Fig. 2** Subgroup analysis of Recurrence for early-stage ovarian cancer: MIS vs OPEN

A subgroup analysis of recurrence rates, excluding three studies that showed significantly shorter follow-up period in MIS


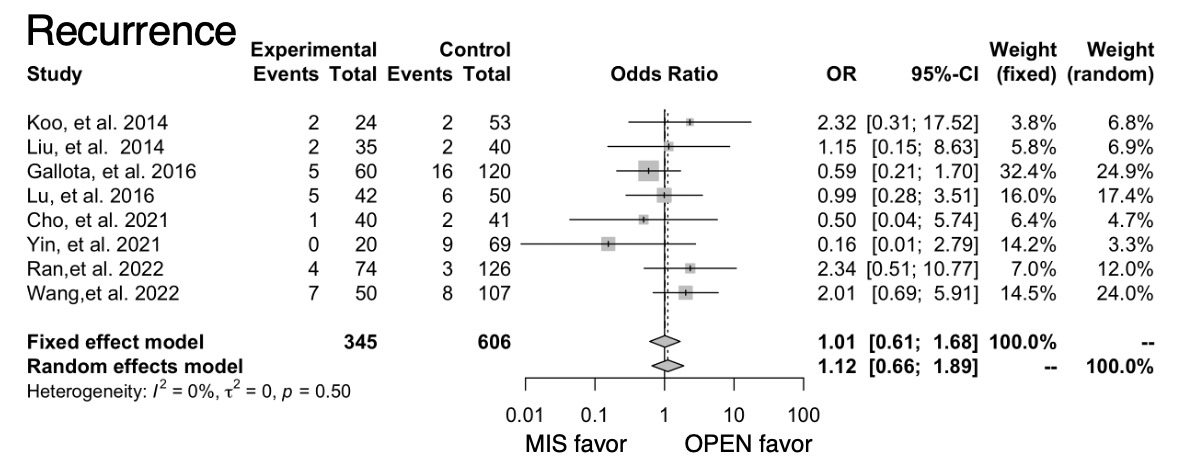

Supplement: Supplementary file 1 — Supplementary file1 (DOCX 319 KB) [file 10147_2023_2320_MOESM1_ESM.docx]
