# Supplementary material for: A single institution’s experience with minimally invasive surgery for ovarian cancer, and a systematic meta-analysis of the literature
Source: Int J Clin Oncol. 2023 Apr 28;28(6):794–803. doi: 10.1007/s10147-023-02320-2 (PMC10232596; doi:10.1007/s10147-023-02320-2)
Supplement: Supplementary file 2 — Supplementary file2 (DOCX 62 KB) [file 10147_2023_2320_MOESM2_ESM.docx]

| **Supplementary Table 1.** Included studies for early-stage ovarian cancer [17, 24-35] | | | | | | | | | |
| --- | --- | --- | --- | --- | --- | --- | --- | --- | --- |
| **Study** | | **Design** | **Period** | **Region** | **MINORS** | **No.** | **Age** | **Histological type** | **Tumor size** |
| Bogani, et al. 2014 | | Retrospective | 1990-2010 | Italy | 17 | MIS 35 | 52±15.8 | All | NA |
|  |  |  |  |  |  | OPEN 32 | 56±16.2 |  | NA |
| Koo, et al. 2014 | | Retrospective | 2006-2012 | Korea | 17 | MIS 24 | 45.8±7.8 | Epithelial | 7.3±4.3^*^ |
|  |  |  |  |  |  | OPEN 53 | 48.4±9.4 |  | 11.2±4.5^*^ |
| Liu, et al. 2014 | | Retrospective | 2002-2010 | China | 15 | MIS 35 | NA | NA | 6.69±1.51^*^ |
|  |  |  |  |  |  | OPEN 40 | NA | NA | 11.50±3.48^*^ |
| Gallota, et al. 2016 | | Retrospective | 2007-2013 | Italy | 14 | MIS 60 | 48 (24-73)^*^ | Epithelial | NA |
|  |  |  |  |  |  | OPEN 120 | 55 (22-81)^*^ |  | NA |
| Lu, et al. 2016 | | Retrospective | 2002-2014 | China | 18 | MIS 42 | 54 (14-69) | Epithelial | NA |
|  |  |  |  |  |  | OPEN 50 | 58 (25-76) |  | NA |
| Minig, et al. 2016 | | Retrospective | 2008-2014 | Spain | 15 | MIS 50 | 52 (44.8-62.3) | Epithelial | 6.8 (5.0-10.0)^*^ |
|  |  |  |  |  |  | OPEN 58 | 52 (44.8-65) |  | 10 (7.8-15.3)^*^ |
| Ditto, et al. 2017 | | Retrospective | 2005-2015 | Italy | 17 | MIS 50 | 46±13.4 | Epithelial | NA |
|  |  |  |  |  |  | OPEN 50 | 42.8±11.2 |  | NA |
| Melamed, et al. 2017 | | Retrospective | 2010-2012 | USA | 17 | MIS 1096 | 57 (49-65) | Epithelial | <1cm 60 (5.4) 1.0-4.9 218 (19.9)  5-9.9 292 (26.6) 10< 382 (34.6) |
|  |  |  |  |  |  | OPEN 1096 | 57 (50-65) |  | <1cm 62 (5.7) 1.0-4.9 222 (20.2)  5-9.9 308 (28.1) 10< 376 (34.3) |
| Merlier, et al. 2020 | | Retrospective | 2000-2018 | France | 16 | MIS 37 | 56.3±16.8 | Epithelial | NA |
|  |  |  |  |  |  | OPEN 107 | 56.2±14.7 |  | NA |
| Cho, et al. 2021 | | Retrospective | 2014-2018 | Korea | 17 | MIS 40 | 46.4 (15-86)^*^ | All | 11 (2.5-28)^*^ |
|  |  |  |  |  |  | OPEN 41 | 53.8 (19-61)^*^ |  | 15.4 (6-40)^*^ |
| Yin, et al. 2021 | | Retrospective | 2010-2020 | China | 15 | MIS 20 | 51 | Clear cell | ≦8cm 29 (32.6) 8< 54 (60.7) ^*^ |
|  |  |  |  |  |  | OPEN 69 | 49 |  | ≦8cm 1 9 (27.5) 8< 48 (69.6) ^*^ |
| Ran, et al. 2022 | Retrospective | | 2012-2020 | Chiina | 18 | MIS 74 | 48.5 (43-54) | Epithelial | 1.0-4.9cm 3 (4.1) 5.0-9.9 21 (28.4)  10.0-14.9 24 (32.4) 15 =< 10 (13.5)  unknown 16 (21.6) |
|  |  |  |  |  |  | OPEN 126 | 48 (44-54) |  | 1.0-4.9cm 5 (4.0) 5.0-9.9 31 (24.6)  10.0-14.9 37 (29.4) 15 =< 38 (30.2)  unknown 15 (11.9) |
| Wang, et al. 2022 | Retrospective | | 2012-2017 | China | 18 | MIS 50 | 35±11.2 | Epithelial | 8 (6-12) |
|  |  |  |  |  |  | OPEN 107 | 38.5±10.9 |  | 8 (6-11) |
| Data are median (range) or mean±SD or n (%) unless otherwise specified. ^*^ Significant difference between MIS and OPEN (P<0.05), NA = not applicable, MINORS = Methodological Index for Nonrandomized Studies | | | | | | | | | |

| **Supplementary Table 1.** Cont | | | | | | | | | | |  |
| --- | --- | --- | --- | --- | --- | --- | --- | --- | --- | --- | --- |
| **Study** | **No.** | **Operative time(minutes)** | **Blood loss(ml)** | **Transfusion** | **Conversion** | **Spillage** | **Up-staging** | **Complication** | | **Hospital day(days)** | **No. of lymph nodes removed** |
|  |  |  |  |  |  |  |  | **Intra-operative** | **Post-operative** |  |  |
| Bogani, et al. 2014 | MIS 35 | 335±74.7^*^ | 300(50-3000) | 1 (2.8)^*^ | 0 | 6 (17.1) | 6 (17.1) | 0 | 1 (2.8)^*^ | 4 (2-30)^*^ | Pelvic 22±5.0^*^  Para-aortic 10±7^*^ |
|  | OPEN 32 | 230±54^*^ | 400(200-1500) | 7 (21.9)^*^ |  | 4 (12.5) | 4 (12.5) | 0 | 9 (28.1)^*^ | 6 (3-14)^*^ | Pelvic 15±9.8^*^  Para-aortic 6±4.6^*^ |
| Koo, et al. 2014 | MIS 24 | 192.9±73.5 | 698±370 | 5 (20.8) | 0 | 13 (54.2) | NA | 0 | 13 (54.2) | 13.7±5.4 | Pelvic 26.8±8.5  Para-aortic 17.7±10.1 |
|  | OPEN 53 | 224.1±85.4 | 973±878 | 15 (28.3) |  | 21 (39.6) | NA | 3 | 30 (47.6) | 13.1±4.1 | Pelvic 27.8±13.2  Para-aortic 21.2±11.2 |
| Liu, et al. 2014 | MIS 35 | 209.7±17.6 | 191.7±98.5 | NA | NA | 3 (8.6) | 6 (17.1) | 0 | 4 (11.4) | 16.3±6.2^*^ | Total 18.23±3.3 |
|  | OPEN 40 | 200.5±20.6 | 345±166 | NA |  | 2 (5.0) | 9 (22.5) | 0 | 5 (12.5) | 21.9±4.9^*^ | Total 19.0±3.2 |
| Gallota, et al. 2016 | MIS 60 | NA | NA | NA | 0 | NA | NA | NA | NA | 3 (2-7)^*^ | Total 16 (2-50) |
|  | OPEN 120 | NA | NA | NA |  | NA | NA | NA | NA | 7 (2-31)^*^ | Total 18 (3-65) |
| Lu, et al. 2016 | MIS 42 | 200 (150-460) | 110 (50-450)^*^ | 1 | NA | NA | 9 (21.4) | 1 (2.4) | 3 (7.1) | 3 (2-14)^*^ | NA |
|  | OPEN 50 | 240 (180-570) | 370 (20-1000)^*^ | 2 |  | NA | 10 (20) | 0 | 4 (8.0) | 7 (3-10^*^) | NA |
| Minig, et al. 2016 | MIS 50 | 225 (180-240) | 200 (200-225)^*^ | 3 (6.0)^*^ | 1 | NA | 12 (24) | 3 | 3 | 2 (1.5-3)^*^ | Pelvic 15 (10.8-21)  Para-aortic10 (4.8-15) |
|  | OPEN 58 | 220 (180-240) | 500 (300-1000)^*^ | 15 (25.7)^*^ |  | NA | 8 (13.8) | 5 | 4 | 5 (4-6.3)^*^ | Pelvic 13.5 (10.8-18.5)  Para-aortic 10 (6-20) |
| Ditto, et al. 2017 | MIS 50 | 207.2±71,6^*^ | 150±57.7^*^ | 0 | 0 | NA | 10 (20) | 0 | 1 (5.0) | 4±2.6^*^ | Pelvic 16.6±7.9  Para-aortic 16.7±6.6 |
|  | OPEN 50 | 180.7±47^*^ | 339.8±225.9^*^ | 4 |  | NA | 13 (26) | 1 (5.0) | 2 (10) | 6.1±1.6^*^ | Pelvic 19.5±9.3  Para-aortic 18.4±9.2 |
| Melamed, et al. 2017 | MIS 1096 | NA | NA | NA | 190 (17.1) | 0 | 81 (12.2%)^*^ | NA | NA | 3 (1-4)^*^ | Total 14 (7-22)^*^ |
|  | OPEN 1096 | NA | NA | NA |  | 0 | 126 (19.2%)^*^ | NA | NA | 4 (3-5)^*^ | Total 12 (6-20)^*^ |
| Merlier, et al. 2020 | MIS 37 | NA | NA | NA | NA | 0 | NA | 3 (13.6) | 7 (20.6) | NA | Pelvic 12 (6-18)^*^  Para-aortic14 (0-23) |
|  | OPEN 107 | NA | NA | NA |  | 4 | NA | 6 (5.7) | 11 (11.1) | NA | Pelvic 7 (0-13)^*^  Para-aortic 9 (0-20) |
| Cho, et al. 2021 | MIS 40 | 201.4 (92-412) | 102 (10-1100)^*^ | 1 (2.5)^*^ | 1 (2.5) | NA | NA | 1 (2.5) | NA | 5 (2-9)^*^ | Total 14.3 (0-46) |
|  | OPEN 41 | 203.0 (92-521) | 372 (10-1870)^*^ | 8 (19.5)^*^ |  | NA | NA | 2 (4.9) | NA | 9.5 (5-20)^*^ | Total 21.1 (0-72) |
| Yin, et al. 2021 | MIS 20 | NA | NA | NA | 12 (60) | NA | NA | NA | NA | NA | NA |
|  | OPEN 69 | NA | NA | NA |  | NA | NA | NA | NA | NA | NA |
| Ran, et al. 2022 | MIS 74 | 269.12±78.98^*^ | 254.46±309.49^*^ | 1 (1.4)^*^ | NA | 19 (29.2)^*^ | NA | NA | 2 (5.3) | 6 (6-8) | NA |
|  | OPEN 126 | 313.08±96.77^*^ | 424.26±293.46^*^ | 9 (7.1)^*^ |  | 12 (10.2)^*^ | NA | NA | 3 (4.7) | 7 (6-9) | NA |
| Wang, et al. 2022 | MIS 50 | 150 (60-300)^*^ | 100 (20-400)^*^ | 0 (0)^*^ | 0 (0) | NA | NA | NA | 3 (6.0) | 6 (3-33)^*^ | Pelvic 26 (8-45)  Para-aortic 5 (1-26) |
|  | OPEN 107 | 180 (150-200)^*^ | 200 (100-1500)^*^ | 8 (7.5)^*^ |  | NA | NA | NA | 10 (9.3) | 8 (2-23)^*^ | Pelvic 26 (8-49)  Para-aortic 7 (1-33) |
| Data are median (range) or mean±SD or n (%) unless otherwise specified. ^*^ Significant difference between MIS and OPEN (P<0.05), NA = not applicable | | | | | | | | | | | |

| **Supplementary Table 1.** Cont | | | | | | |
| --- | --- | --- | --- | --- | --- | --- |
| **Study** | **No.** | **Follow up period(month)** | **Reccurence** | **Port-site metastasis** | **OS** | **PFS** |
| Bogani, et al. 2014 | MIS 35 | 64 (37-106)^*^ | 4 (11.4) | NA | 94.3% | NA |
|  | OPEN 32 | 100 (61-287)^*^ | 9 (28.1) |  | 84.4% | NA |
| Koo, et al. 2014 | MIS 24 | 31.7±20.7 | 2 (8.3) | 0 | 3-year OS 86.1% | 91.6% |
|  | OPEN 53 | 31.1±19.1 | 2 (3.8) |  | 3-year OS 94.7% | 96.2% |
| Liu, et al. 2014 | MIS 35 | 36-84 | 2 (5.71) | 0 | 3-year OS 97.1% | NA |
|  | OPEN 40 | 36-84 | 2 (5.0) |  | 3-year OS 97.5% | NA |
| Gallota, et al. 2016 | MIS 60 | 38 (24-48) | 5 (8.3) | 0 | 4-year OS 92% | 3-year PFS 89% |
|  | OPEN 120 | 38 (24-48) | 16 (13.3) |  | 4-year OS 91% | 3-year OS 81% |
| Lu, et al. 2016 | MIS 42 | 82 (16-152) | 5 (12.0) | 0 | 5-year OS 91.3% | NA |
|  | OPEN 50 | 82 (16-152) | 6 (12.0) |  | 5-year OS 88.4% | NA |
| Minig, et al. 2016 | MIS 50 | 25.9 (11.2-38.5)^*^ | 6 (12.0) | NA | 85.4% | 73.6% |
|  | OPEN 58 | 34.3 (28.4-47.8)^*^ | 7 (12.1) |  | 67.0% | 64.8% |
| Ditto, et al. 2017 | MIS 50 | 49.5±64^*^ | NA | 0 | HR 0.87  (95%CI : 0.08-9.19) | HR 0.79  (95%CI : 0.31-2.01) |
|  | OPEN 50 | 52.6±31.7^*^ | NA |  |  |  |
| Melamed, et al. 2017 | MIS 1096 | 28.7 (20.4-38.9) | NA | NA | HR 0.77  (95%CI : 0.54-1.09) | NA |
|  | OPEN 1096 | 29.3 (20.6-39.3) | NA |  |  | NA |
| Merlier, et al. 2020 | MIS 37 | 24 (11-50)^*^ | 2 (5.4) | NA | Adjusted HR 0.28  (95%CI : 0.04-2.13) | Adjusted HR 0.36  (95%CI : 0.11-1.19) |
|  | OPEN 107 | 42 (26-66)^*^ | 31 (29.0) |  |  |  |
| Cho, et al. 2021 | MIS 40 | 35.8 (15-72) | 1 (2.5) | NA | 100% | 97.5% |
|  | OPEN 41 | 48.2 (1-74) | 2 (4.9) |  | 100% | 95.1% |
| Yin, et al. 2021 | MIS 20 | Median 42.6 | 0 | NA | 5-year OS 100% | 2-year PFS 100% |
|  | OPEN 69 | Median 36.5 | 9 |  | 5-year OS 91.9% | 2-year OS 90.1% |
| Ran, et al. 2022 | MIS 74 | 43 (38.8-47.2) | 4 (5.4) | NA | 100% |  |
|  | OPEN 126 | 45 (36.0-54.0) | 3 (2.4) |  | 96% |  |
| Wang, et al. 2022 | MIS 50 | 63.6±27.6 | 7 (14) | NA | 100% | 5-year PFS 100% |
|  | OPEN 107 | 61.8±28.2 | 8 (7.5) |  | 97.2% | 5-year PFS 98.8% |
| Data are median (range) or mean±SD or n (%) unless otherwise specified. ^*^ Significant difference between MIS and OPEN (P<0.05), NA = not applicable | | | | | |  |

| **Supplementary Table 2.** Included studies for advanced-stage ovarian cancer [8, 36-41] | | | | | | | |  |  |
| --- | --- | --- | --- | --- | --- | --- | --- | --- | --- |
| **Study** | **Design** | **Period** | **Region** | **MINORS** | **No.** | **Age** | **Histological type** | **Tumor size** | **Response after NAC** |
| Favero, et al. 2015 | Prospective | 2011-2014 | Brazil | 19 | MIS 10 | 58 (42-73) | High grade serous | NA | NA |
|  |  |  |  |  | OPEN 11 | 61.3 (41-80) |  | NA | NA |
| Gueli Alletti, et al. 2016 | Retrospective | 2013-2014 | Italy | 17 | MIS 30 | 59 (48-80) | Epithelial | NA | CR 6 (20)  PR 24 (80) |
|  |  |  |  |  | OPEN 65 | 62 (40-81) |  | NA | CR 12 (18.5)  PR 53 (81.5) |
| Ceccaroni, et al. 2016 | Retrospective | 2007-2015 | Italy | 18 | MIS 21 | 56 (34-79) | Epithelial | NA | NA |
|  |  |  |  |  | OPEN 45 | 60 (34-80) |  | NA | NA |
| Melamed, et al. 2017 | Retrospective | 2010-2012 | USA | 17 | MIS 450 | 63.9±11.7 | Epithelial | NA | NA |
|  |  |  |  |  | OPEN 2621 | 63.2±11.1 |  | NA | NA |
| Brown, et al. 2019 | Retrospective | 2006-2017 | USA | 15 | MIS 53 | 66.6±11.0 | Epithelial | 4.3±5.6 | CR 9 (17.0)  PR 43 (81.1)  PD 1 (1.9) |
|  |  |  |  |  | OPEN 104 | 67.1±9.6 |  | 4.9±6.6 | CR 13 (12.5)  PR 81 (77.9)  SD 6 (5.8)  PD 2 (1.9)  no info 2 (1.9) |
| Liang, et al. 2017 | Retrospective | 2007-2016 | China | 16 | MIS 64 | 53.5±11.4 | All | 7.9±4.6^*^ | NA |
|  |  |  |  |  | OPEN 68 | 51.9±11.8 |  | 10.8±5.2^*^ | NA |
| Lecointre, et al. 2022 | Retrospective | 2009-2019 | Italy | 16 | MIS 37 | 62.8±11.2 | Serous | NA | NA |
|  |  |  |  |  | OPEN 40 | 61.7±9.5 |  | NA | NA |
| Data are median (range) or mean±SD or n (%) unless otherwise specified. ^*^ Significant difference between MIS and OPEN (P<0.05), NA = not applicable,  MINORS = Methodological Index for Nonrandomized Studies | | | | | | | | |  |

| **Supplementary Table 2. Cont** | | | | | | | | | | | | | |
| --- | --- | --- | --- | --- | --- | --- | --- | --- | --- | --- | --- | --- | --- |
| **Study** | **No.** | **Operative time(minutes)** | **Blood loss(ml)** | **Transfusion** | **Conversion** | **Complication** | | **Hospital day(days)** | **Time to adjuvant chemotherapy(days)** | **No. of lymph nodes removed** | **Residual disease** | |  |
|  |  |  |  |  |  | **Intra operative** | **Post operative** |  |  |  |  |  |  |
| Favero, et al. 2015 | MIS 10 | 292 (214-360) | 180 (92-320) | 0 | 0 | 0 | 2 (20.0) | 3.6 (2-12) | NA | Pelvic 8 (3-31)  Para-aortic 6 (2-12) | Optimal 10 (100) | |  |
|  | OPEN 11 | 264 (192-410) | 292 (215-360) | 2 (18.2) |  | 0 | 1 (9.1) | 5.4 (3-14) | NA |  | Optimal 11 (100) | |  |
| Gueli Alletti, et al. 2016 | MIS 30 | 285 (124-418)^*^ | 100 (50-200)^*^ | NA | NA | NA | 0 | 2 (2-3)^*^ | 20 (10-30)^*^ | NA | No residue 29 (96.6) | |  |
|  | OPEN 65 | 180 (120-450)^*^ | 200 (100-400)^*^ | NA |  | NA | 2 (3.1) | 4 (3-8)^*^ | 35 (19-60)^*^ | NA | No residue 62 (95.4) | |  |
| Ceccaroni, et al. 2016 | MIS 21 | 280 (15-420) | 250 (100-500)^*^ | NA | 0 | 0^*^ | 4 (19.0)^*^ | 9 (5-25)^*^ | 15 (10-30)^*^ | NA | No residue 20 (95.2) | |  |
|  | OPEN 45 | 330 (140-660) | 650 (100-4500)^*^ | NA |  | 8 (17.8)^*^ | 21 (46.7)^*^ | 15 (6-50)^*^ | 28 (20-35)^*^ | NA | No residue 38 (84.4) | |  |
| Melamed, et al. 2017 | MIS 450 | NA | NA | NA | 72 (16) | NA | NA | 4 (2-6)^*^ | NA | NA | No residue 130 (46.9)  Optimal 90 (32.5)  Suboptimal 57 (20.6) | |  |
|  | OPEN 2621 | NA | NA | NA |  | NA | NA | 5 (4-7)^*^ | NA | NA | No residue 789 (49.5)  Optimal 445 (27.9)  Suboptimal 361 (22.6) | |  |
| Brown, et al. 2019 | MIS 53 | 171±47^*^ | 156±151^*^ | 1 (1.9)^*^ | NA | 1 (1.9) | 38 (71.7) | 3±2.6^*^ | NA | NA | No residue 32 (60.4)^*^  Optimal 19 (35.8)^*^  Suboptimal 2 (3.8)^*^ | |  |
|  | OPEN 104 | 150±55^*^ | 278±277^*^ | 17 (15.9)^*^ |  | 3 (2.9) | 78 (75.0) | 5.7±2.8^*^ | NA | NA | No residue 44 (42.3)^*^  Optimal 42 (40.4)^*^  Suboptimal 18 (17.3)^*^ | |  |
| Liang, et al. 2017 | MIS 64 | 304±111.7^*^ | 232.2±295.0^*^ | NA | 2 (4.2) | 3 (4.7) | 5 (7,8) | NA | 10.8±6.9^*^ | Pelvic 15.6±7,9  Para-aortic 5.7±5.0 | No residue 55 (85.9)  Optimal 6 (9.4)  Suboptimal 3 (4.7) | |  |
|  | OPEN 68 | 389±117.4^*^ | 796.6±613.2^*^ | NA |  | 4 (5.9) | 11 (16.2) | NA | 20.0±18.6^*^ | Pelvic 17.0±8.4  Para-aortic 4.6±3.7 | No residue 46 (67.6)  Optimal 6 (8.8)  Suboptimal 16 (23.5) | |  |
| Lecointre, et al. 2022 | MIS 37 | 312±96 | 376.9±411.7^*^ | 13 (35.1)^*^ | 1 (2.7) | 6 (16.2) | 7 (18.9)^*^ | 7.6±5.7^*^ | 31.6±17.4 | Total 29.6±9.7 | No residue 37 (100) | |  |
|  | OPEN 40 | 294±90 | 613±434.7^*^ | 28 (70)^*^ |  | 5 (12.5) | 21 (52.5)^*^ | 12.1±6.2^*^ | 30.7±24.4 | Total 34.1±14.8 | No residue 40 (100) | |  |
| Data are median (range) or mean±SD or n (%) unless otherwise specified. ^*^ Significant difference between MIS and OPEN (P<0.05), NA = not applicable | | | | | | | | | | | |  | |

| **Supplementary Table 2. Cont** | | | | | | |
| --- | --- | --- | --- | --- | --- | --- |
| **Study** | **No.** | **Follow up period(month)** | **Reccurence** | **Port-site metastasis** | **OS** | **PFS** |
| Favero, et al. 2015 | MIS 10 | Median 20^*^ | 8 (80) | 0 | 80% | NA |
|  | OPEN 11 | Median 34^*^ | 10 (88) |  | 100% | NA |
| Gueli Alletti, et al. 2016 | MIS 30 | Median 28 | NA | NA | NA | 18 month |
|  | OPEN 65 | Median 28 | NA |  | NA | 12month |
| Ceccaroni, et al. 2016 | MIS 21 | 47.3 (12-72) | 7 (33.3) | NA | NA | 42.3 month |
|  | OPEN 45 | 52.5 (5-117) | 16 (35.6) |  | NA | 45.2 month |
| Melamed, et al. 2017 | MIS 450 | Median 32 | NA | NA | Adjusted HR 1.09  (95%CI: 0.93-1.28) | NA |
|  | OPEN 2621 | Median 32 | NA |  |  | NA |
| Brown, et al. 2019 | MIS 53 | NA | NA | NA | 37 months  HR 0.92  (95%CI: 0.54-1.55) | 27 months  HR 0.83  (95%CI: 0.51-13.4) |
|  | OPEN 104 | NA | NA |  | 35 months | 29 months |
| Liang, et al. 2017 | MIS 64 | 18 (5-122) | NA | NA | 3-year OS 88.7% | 3-year PFS 55.5% |
|  | OPEN 68 | 18 (5-122) | NA |  | 3-year OS 83.7% | 3-year PFS 33.3% |
| Lecointre, et al. 2022 | MIS 37 | Median 24.9 | NA | NA | 23.1 months | 14.8 month |
|  | OPEN 40 | Median 24.9 | NA |  | 26.3 months | 12 months |
| Data are median (range) or mean±SD or n (%) unless otherwise specified. ^*^ Significant difference between MIS and OPEN (P<0.05), NA = not applicable | | | | | | |
